# Myalgic Encephalomyelitis/Chronic Fatigue Syndrome in the Era of the Human Microbiome: Persistent Pathogens Drive Chronic Symptoms by Interfering With Host Metabolism, Gene Expression, and Immunity

**DOI:** 10.3389/fped.2018.00373

**Published:** 2018-12-04

**Authors:** Amy Proal, Trevor Marshall

**Affiliations:** Autoimmunity Research Foundation, Thousand Oaks, CA, United States

**Keywords:** microbiome, dysbiosis, holobiont, pathobiont, autoantibody, ME/CFS, molecular mimicry, gut-brain axis

## Abstract

The illness ME/CFS has been repeatedly tied to infectious agents such as Epstein Barr Virus. Expanding research on the human microbiome now allows ME/CFS-associated pathogens to be studied as interacting members of human microbiome communities. Humans harbor these vast ecosystems of bacteria, viruses and fungi in nearly all tissue and blood. Most well-studied inflammatory conditions are tied to dysbiosis or imbalance of the human microbiome. While gut microbiome dysbiosis has been identified in ME/CFS, microbes and viruses outside the gut can also contribute to the illness. Pathobionts, and their associated proteins/metabolites, often control human metabolism and gene expression in a manner that pushes the body toward a state of illness. Intracellular pathogens, including many associated with ME/CFS, drive microbiome dysbiosis by directly interfering with human transcription, translation, and DNA repair processes. Molecular mimicry between host and pathogen proteins/metabolites further complicates this interference. Other human pathogens disable mitochondria or dysregulate host nervous system signaling. Antibodies and/or clonal T cells identified in patients with ME/CFS are likely activated in response to these persistent microbiome pathogens. Different human pathogens have evolved similar survival mechanisms to disable the host immune response and host metabolic pathways. The metabolic dysfunction driven by these organisms can result in similar clusters of inflammatory symptoms. ME/CFS may be driven by this pathogen-induced dysfunction, with the nature of dysbiosis and symptom presentation varying based on a patient's unique infectious and environmental history. Under such conditions, patients would benefit from treatments that support the human immune system in an effort to reverse the infectious disease process.

## Introduction: ME/CFS Enters the Era of the Human Microbiome

Toward the end of a career spent studying persistent bacteria in chronic disease, microbiologist Gerald Domingue wrote, “It is unwise to dismiss the pathogenic capacities of any microbe in a patient with a mysterious disease” (1). This thinking greatly applies to the illness ME/CFS. ME/CFS is characterized by neuroinflammation, severe fatigue, excessive post-exertional exhaustion, disturbed sleep, flu-like episodes, cognitive problems, sensory hypersensitivity, muscle and joint pain, headache, bowel symptoms, and severe impairment of daily functioning (2). Severely ill individuals are often wheelchair dependent, bedridden, and unable to perform basic tasks of work or daily living.

The history of ME/CFS strongly suggests that infectious agents play a central role in driving the disease process. These include early associations with Epstein Barr Virus (EBV)/Human Herpes Virus 6 (HHV6), the relapsing-remitting nature of ME/CFS symptoms and antibodies/“autoantibodies” detected in patients with the disease (3, 4). A number of ME/CFS outbreaks have also been reported, in which numerous people in the same geographical location developed the illness simultaneously (2). Indeed, many ME/CFS patients present with symptoms after suffering from a severe bacterial or viral infection. These infections often correlate with travel to a foreign country or exposure to pollutants or molds, suggesting that such pathogens take advantage of factors that compromise the host immune system.

Chronic inflammation is a hallmark of persistent infection. Reports of cytokine activation in ME/CFS clarify that the disease is associated with an inflammatory response (5). Montoya et al. (6) found ME/CFS cytokine activation increased with disease severity, suggesting patients may struggle with a growing infectious burden over time. Other ME/CFS research teams have identified various forms of mitochondrial dysfunction in patients with the illness (7). There are now dozens of well-characterized mechanisms by which bacteria and viruses dysregulate mitochondrial metabolism (8, 9).

Many research teams have searched for well-characterized single pathogens in patients with ME/CFS. These analyses often reveal elevated titers of IGG and IGM antibodies toward pathogens such as Epstein Barr Virus, Cytomegalovirus, Parvovirus 19 and *M. pneumonia* (2, 3, 10). Several teams have also attempted to identify a single novel pathogen that might drive the entire ME/CFS disease process. However, the discovery of the human microbiome now allows single microbes and viruses to be studied as members of complex communities. Humans harbor these vast ecosystems of bacteria, viruses and fungi in nearly all tissue and blood (11–14). Organisms in the microbiome continually interact with each other, and with the human genome, to regulate host metabolism and gene expression in both health and disease (15, 16).

A growing number of inflammatory disease states, including neurological conditions and cancers, are tied to dysbiosis or imbalance of these human microbiome communities (17–20). Gut microbiome dysbiosis has been identified in ME/CFS (21). This dysbiosis is characterized by changes in microbe species composition and/or diversity. Pathogens, or groups of pathogens, can promote dysbiosis by altering their gene expression in ways that promote virulence, immunosuppression and dysregulation of host genetic and metabolic pathways (22).

When seemingly disparate biomedical findings on ME/CFS are interpreted through the lens of these microbiome-based paradigms and platforms, a cohesive picture of the ME/CFS disease process emerges. ME/CFS may be driven by pathogen-induced dysfunction, with resulting microbiome dysbiosis varying based on a patient's unique infectious and environmental history. Under such conditions, patients would benefit from treatments that, like those now being developed for cancer, support the human immune system in an effort to reverse the inflammatory disease process.

## The Human Microbiome Persists Throughout the Body

In the USA, ME/CFS cases were first formally reported to the CDC in the 1980s (2). At the time, human microbes were typically only detected with culture-based laboratory methods. Then, around the year 2000, novel genome-based technologies began to revolutionize the field of microbiology (23, 24). These technologies identify microbes based on their DNA or RNA signatures rather than their ability to grow in the laboratory. The results of these genome-based analyses were remarkable: vast communities of microbes were identified in the human body that had been missed by the older culture-based techniques. These extensive ecosystems of bacteria, viruses, fungi, and archaea are collectively known as the human microbiome (25–27).

Today, so many novel microbes have been identified in *Homo sapiens* that our human cells are equivalent to or even outnumbered by those of our microbial inhabitants (28). The tens of millions of unique genes harbored by this microbiome dwarf the ~20,500 genes in the human genome (12, 29). For example, just one 2017 analysis of the human gut, skin, mouth, and vaginal microbiomes uncovered millions of previously unknown microbial genes (11). This has forced science to redefine the human condition. Humans are best described as holobionts, in which the microbial genomes and the human genome continually interact to regulate metabolism and immunity (15, 16).

Early human microbiome studies characterized microbial ecosystems in the gut and on mucosal surfaces. However, the microbiome has now been shown to extend to nearly every human body site. These include the lungs, the bladder, the placenta, the testes, and the uterus [(19, 30–33)]. Jakobsen et al. (35) found that previously sterile implants removed from joints, bones, pacemakers, and skulls of symptom-free patients were colonized by a range of bacterial and fungal organisms. Another study demonstrated the presence of novel tissue specific bacterial DNA profiles in a variety of mouse organs including the brain, heart, liver, muscle and adipose tissue (36).

Microbial communities also appear to persist in healthy human blood (37–39). A DNA virome was recently identified in healthy human blood (40). Another study reported both bacterial and fungal communities in the blood of healthy subjects. Analysis of these organisms was performed by microbial resuscitation of blood culturing and microscopy in addition to next generation DNA sequencing (41). Whittle et al. (42) recently characterized a human blood microbiome using a range of complementary molecular and classical molecular biology techniques. Another study identified a larger amount of bacterial rDNA in blood specimens from healthy individuals than in matched reagent controls (24).

Kowarsky et al. (14) detected over 3,000 previously unidentified viruses, bacteria, and fungi in human blood samples obtained from immunocompromised patients. The study almost doubled the total number of anelloviruses found in humans. In order to classify many of these organisms the team was forced to add new branches to the “tree of life.” They concluded that the newly discovered microbes “may prove to be the cause of acute or chronic diseases that, to date, have unknown etiology.”

The microbiome is inherited and evolves with the host. Babies are seeded in the womb, during birth, and after birth by extensive microbiome communities in the placenta, the vaginal canal, and breast milk, among other body sites (43, 44). Microbes/pathogens acquired from the external environment are further incorporated into the microbiome over time. For example, once acquired, Cytomegalovirus persists as a member of the microbiome—with a significant impact on host immunity. Brodin et al. (45) found that the lifelong need for the body to control CMV causes approximately 10% of all T cells in CMV+ individuals to be directed against the virus.

Immune cells and associated microbes can travel between the human body and brain via several newly discovered pathways that bypass the classical blood-brain barrier (46). Benias et al. (47) documented a previously uncharacterized fluid-filled lattice of collagen bundles that appears to connect all human tissues. This human interstitium drains directly into the lymph nodes. Two research teams have demonstrated the existence of a previously undiscovered meningeal lymphatic system (46, 48, 49). The network's fluid pathways connect the cerebrospinal fluid and cervical lymph nodes directly to the brain.

## Our Understanding of the Human Microbiome and Virome Continues to Evolve

While great progress has been made in characterizing the human microbiome, our understanding of the body's microbial ecosystems is still in its infancy. Metagenomic analyses of the microbiome in all body sites regularly identify species or strains of bacteria, archaea, fungi, and/or viruses not previously understood to persist in *Homo sapiens* (13). For example, just one study of the bladder microbiome identified 129 previously unidentified viruses in subjects' urine samples (50).

New strains of known microbes are also regularly identified. For example, as of August 2018, the NCBI database contains ~1,833 *Lactobacillus* genomes, with newly characterized *Lactobacillus* genomes added on a weekly basis (51). Successful identification of these known or novel human organisms hinges on the careful choice of technology and methodology used for detection purposes (26). While the majority of human microbiome studies center on bacteria, awareness of viruses, fungi, and archaea has increased in recent years (52). For example, Manuela et al. (53) found an almost 1:1 ratio of archaeal to bacterial 16S rRNA genes in human appendix and nose samples. Identification of this archaeome abundance and diversity required use of a very specific archaea-targeting methodology.

Viruses are the most abundant life forms on the planet and in the human body, but have been relatively hard to detect until very recently (54). Viruses that primarily infect bacteria, called bacteriophages, are particularly abundant in human microbiome communities. Nguyen et al. (55) estimate that ~31 billion bacteriophages traffic human tissue and blood on a daily basis. However, the human virome also harbors a plethora of human-associated viruses. Newberry et al. (56) are correct to assert that this human virome is understudied in ME/CFS.

**Figure 1:**
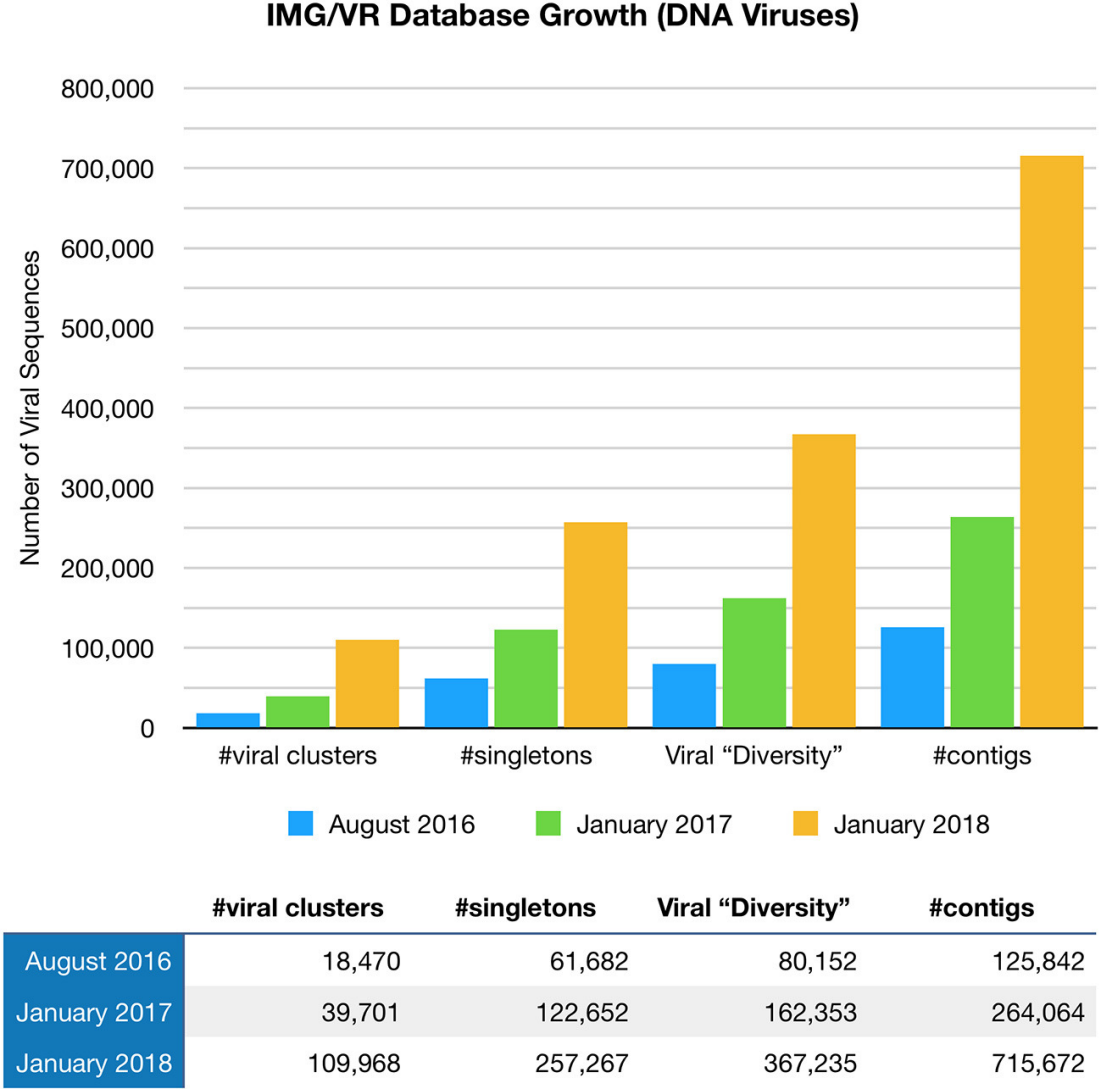
The IMG/VR database catalogs viruses in Earth's ecosystems including the human body. Viral diversity in IMG/VR has more than tripled since August 2016.

Paez-Espino et al. (57) at the Joint Genome Institute have undertaken a large project called “Uncovering the Earth's Virome.” The Project's goal is to better identify known and novel viruses in Earth's ecosystems including the human body. Viral identification requires the use of specific metagenomic tools, pipelines, and annotation platforms, with findings entered in the JGI IMG/VR Database (57). The Project is progressing at such a rapid pace that viral diversity in IMG/VR has more than tripled since August 2016 (58), (Figure 1). Nevertheless, the vast majority of the gene content (over 15 million genes in total) remains unknown or hypothetical.

We must subsequently consider the possibility that as-yet unidentified microorganisms may contribute to chronic inflammatory conditions like ME/CFS. Failure to do so would be akin to studying ~2% of the animals in the rainforest and arriving at firm conclusions about the entire ecosystem based on that information alone. Certain members of the human microbiome may also be difficult to detect based on their location and/or lifestyle. For example, Chen et al. (59) found that elevated cytokine expression in response to HSV-infected peripheral nerve ganglia persisted even when the virus entered a latent, non-replicating state.

While it is important to pursue identification of novel organisms in ME/CFS blood and tissue, ample data already exists on better-studied components of the microbiome. Microbial and viral survival strategies, virulence mechanisms and collective behaviors are also characterized by a high degree of functional redundancy (60, 61). We must accept the complexity inherent to the human microbiome and further study these common mechanisms of survival and persistence. We must examine microbe and viral activity, microbe and viral gene expression, and the myriad ways in which the proteins and metabolites created by these organisms interact with the host immune system, the host genome, and each other.

## The Human Holobiont: Microbes and Their Metabolites Modulate the Activity of Human Pathways

The genes of our microbial inhabitants greatly outnumber the ~20,500 in the human genome. It follows that the majority of metabolites in *Homo sapiens* are produced or modified by the microbiome. Wikoff et al. (62) found a large effect of the gut microbiome on murine blood metabolites including antioxidants, toxins and amino acids. For example, production of the metabolite indole-3-propionic acid was completely dependent on the presence of gut microbes and could be established by colonization with the bacterium *Clostridium sporogenes*.

Many microbes, viruses and their corresponding proteins/metabolites directly modulate the activity of host metabolic, immune, and neurological pathways. In other words, the human holobiont is controlled by the human genome, our microbial/viral genomes and their respective metabolites working in tandem. A growing number of studies provide examples of this metabolic overlap. While many such studies have been conducted in mice, their general trends carry over to humans.

For example, ME/CFS is associated with natural killer (NK) cell abnormalities, including reduced natural killer cell activity (63). These findings must be interpreted to account for the fact that NK activity is modulated by the bacterial microbiome. One study found that bile acids modified by the gut microbiome impacted liver cell gene expression in a manner that controlled NK cell accumulation and anti-tumor activity (64). Similarly, a mixture of lactic acid bacteria from kefir increased the cytotoxicity of human NK KHYG-1 cells to human chronic leukemia cells and colorectal tumor cells (65). The microbiome and its metabolites also impact the activity of related immune cells. Rothhammer et al. (66) found that tryptophan created by the gut microbiome interacted with the AHR receptor on microglia/astrocytes. Subsequent changes in gene expression regulated communication between the two cell types.

Various forms of autonomic dysfunction are also common in ME/CFS (67, 68). It is subsequently important to consider that the microbiome may contribute to host blood pressure regulation. Pluznick et al. (69) found that gut microbiome-derived Short Chain Fatty Acids such as acetate and propionate travel to the kidneys and blood vessels. There they impacted activity of Olfr78 and Gpr41, two host receptors that control circulation and blood flow.

## Pathogens and Their Proteins/Metabolites Can Dysregulate Human Genetic and Metabolic Pathways

Microbial modulation of host pathways can also drive inflammatory disease processes. Pathogens and their associated proteins/metabolites control human metabolism and gene expression in a manner that can push the holobiont toward a state of imbalance and illness. For example, Rizzo et al. (70) found that Human Herpes Viruses 6A/6B infected NK cells. This infection significantly modified expression of key host miRNAs and transcription factors. *Mycobacterium leprae* has been shown to alter human gene expression in a manner that allows it to hijack and reprogram adult Schwann cells to a stem-like state (71). In a murine model of diabetes, Liu et al. (72) found that eLtaS, a protein created by *S. aureus*, prevented insulin from correctly binding its target receptor. This inhibited the phosphorylation of downstream signaling proteins and caused the mice in the study to develop impaired glucose tolerance.

The ability of pathogens to interfere with host metabolism is tied to the dynamics of the communities in which they persist. Like organisms in any ecosystem, human microbes constantly interact, both directly and indirectly. The proteins and metabolites they create are also in continual interplay. Communities of microbes often exhibit synergistic interactions for improved nutrient acquisition, protection from host defenses, and survival in an inflammatory environment (73). These include biofilm formation and cooperative signaling via quorum sensing peptides. Humphries et al. (74) recently reported that biofilm bacteria can additionally communicate via ion channel-mediated electrical signaling.

Even viruses seldom act as single entities. Diversity and equilibrium of the bacterial microbiome is regulated by bacteriophage predator-prey dynamics (75). Pfeiffer and Virgin (27) found that enteric viral virulence is regulated by the activity of neighboring bacteria, fungi, and even helminths. These processes are called “transkingdom interactions.” For example, human norovirus can bind carbohydrate histo-blood group antigens present on certain bacterial cells. This facilitates the ability of norovirus to infect human B cells (76).

## Microbiome Dysbiosis

Interacting microbes may contribute to dysbiosis or imbalance of microbiome communities (77). This dysbiosis is characterized by substantial shifts in community structure and diversity. In many cases, pathogens proliferate to inhabit niches once occupied by more innocuous microbes.

Most well-studied inflammatory disease states are tied to some form of microbiome dysbiosis. These include psoriatic arthritis, systemic lupus erythematosus, type 1 and 2 diabetes, Parkinson's disease and a growing number of cancers (34, 78, 79). The gut microbiome can initiate and promote colorectal cancer at all stages of tumorigenesis by acting as an inducer of DNA damage, generating epigenetic changes, regulating cell growth, and modulating host immune responses (80). The breast tissue microbiome of women with breast cancer has been shown to differ substantially in composition, virulence and diversity from that of healthy controls (81). Species composition of the bronchoalveolar microbiome shifted toward a more pathogenic state in patients with sarcoidosis (82). Alterations in the enteric virome were reported prior to disease onset in children susceptible to developing type 1 diabetes (83).

Several research teams have tied ME/CFS to bacterial gut microbiome dysbiosis. Giloteaux et al. (21) found that gut microbiome bacterial diversity was decreased in ME/CFS subjects compared to heathy controls. The team also noted increases in certain bacterial species associated with either pro-inflammatory or anti-inflammatory activity. Nagy-Szakal et al. (84) also analyzed the ME/CFS gut microbiome. The study detected seven gut bacterial species whose relative abundance differed from that of control subjects and were strongly associated with ME/CFS.

While these findings are of interest, gut microbiome composition is additionally impacted by a host of environmental variables that cause large shifts in the region's microbial ecosystems. These include geographic location, food consumption, and even time of day (85). Many research teams studying inflammatory conditions have struggled to isolate and/or replicate disease-induced gut microbiome dysbiosis in the face of this “noise.” For example, Frémont et al. (86) found that intestinal microbiome species composition differed between patients with ME/CFS and healthy controls. However, significant changes in intestinal microbiome composition were also identified between control subjects from Norway and controls subjects from Belgium. This variation was proposed to arise from differences in diet between the two cultures.

Studies of the ME/CFS blood microbiome may be less subject to this environment-induced variability. Furthermore, identification of microbial and/or viral communities in ME/CFS blood would allow for a broader picture of possible infectious and inflammatory processes. For example, Giloteaux et al. (21) found that ME/CFS subjects had higher levels of bacterial lipopolysaccharides (LPS), LPS-binding proteins and soluble CD14 in blood. The team suggested that these inflammatory markers may indicate translocation of gut bacteria into the blood. However, the markers could also reflect the presence of bacteria in the blood itself.

The blood microbiome can be characterized if the microbial DNA/RNA in samples is first separated from that derived from the human genome. Olde Loohuis et al. (87) used RNA sequencing of reads from whole blood to analyze microbial communities in the blood of almost 200 patients with three neurological conditions: bipolar disorder, schizophrenia, and amyoytrophic lateral sclerosis. The team identified a wide range of bacterial and archaeal phyla in subjects with all three disease states. They observed increased microbial diversity in schizophrenia subjects compared to the two other groups, and replicated the finding in an independent dataset. Stephen Quake's cell-free DNA shotgun sequencing technologies can also characterize bacterial communities in human blood, and can be additionally extended to identify viruses and fungi (14).

Communities of microbes and viruses may also persist in ME/CFS brain tissue. Readhead et al. (88) recently detected a range of persistent viruses in the Alzheimer's brain. These included herpesviruses, torque teno viruses, adenoviruses, and coronaviruses. The Alzheimer's brain has also been shown to harbor bacterial and fungal communities (89, 90). Branton et al. (91) identified hundreds of bacteria and bacteriophage-derived samples in brain tissue removed from patients with epilepsy, and in brain samples obtained from HIV/AIDS patients after autopsy.

In fact, studies of the virome provide significantly extended context on microbiome community dynamics and disease processes. This is because bacteriophages (phages) infect, and subsequently modulate the activity of the bacterial microbiome (75). For example, Duerkop et al. (92) characterized the intestinal virome in a model of T-cell-mediated murine colitis. The intestinal phage population changed in colitis, and transitioned from an ordered state to a stochastic dysbiosis. Phage populations that expanded during colitis were frequently connected to bacterial hosts that benefit from or are linked to intestinal inflammation. Tetz et al. (93) identified changes in the Parkinson's gut bacteriophage community. These included shifts in the phage/bacteria ratio of bacteria known to produce dopamine.

Species-level studies of the microbiome are also greatly enhanced by analyses that provide further context on disease activity. This is an important consideration because, in theory, the microbiome of a patient with ME/CFS could harbor the exact same microbial, viral and/or fungal species as that of a healthy subject. Yet many of these organisms could be *acting* very differently in patients with the disease. Species-level analyses of the ME/CFS microbiome must subsequently be accompanied by studies that characterize microbial and viral gene expression and/or metabolism.

## Composition of the Human Proteome and Metabolome Reflect Microbiome and Activity

Since many of the proteins and metabolites in the human holobiont are microbial in origin, composition of the ME/CFS proteome and metabolome change with microbiome species composition. Proteome and metabolome analyses additionally reflect microbiome activity. This is because microbes and viruses frequently alter their gene expression in ways that cause them to express different proteins and metabolites over time. The human genome and related epigenetic changes also contribute to the metabolic diversity, although the high level of redundancy between human and microbial metabolites can make the origin of these associations hard to pinpoint.

**Figure 2:**
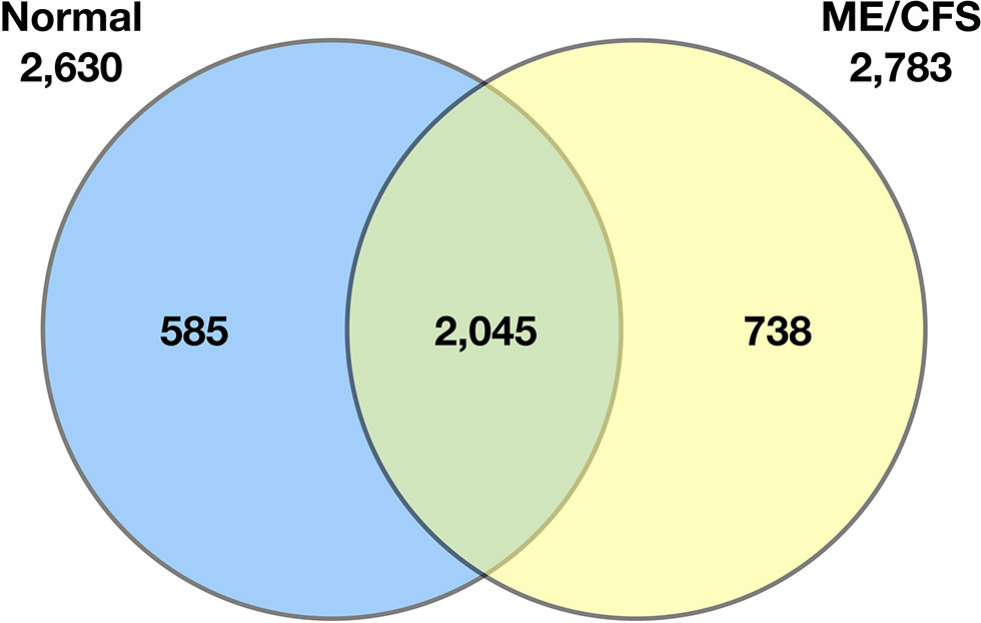
Venn diagram of the qualitative distribution of proteins identified in cerebrospinal fluid from normal control subjects and ME/CFS subjects. Seven hundred and thirty eight of 2,783 identified proteins (26.5%) were unique to patients with ME/CFS. The numbers of proteins for each category separately is shown outside the circles (2,630 for normal controls, 2,783 for ME/CFS) (94).

Schutzer et al. (94) demonstrated that the ME/CFS cerebrospinal proteome differs substantially from that of healthy controls (Figure 2). Indeed, 738 of 2,783 identified proteins (26.5%) were unique to patients with ME/CFS, providing strong evidence that ME/CFS is indeed characterized by microbiome dysbiosis in tissue and blood. Composition of the blood metabolome has also been shown to shift in ME/CFS. One such study reported elevated plasma levels of choline, carnitine, and complex lipid metabolites in ME/CFS patients (84). Another analysis demonstrated a sustained hypo-metabolic response in patients with the disease (95). This dour-like state can be driven by exposure to adverse environmental conditions, as would be expected if the ME/CFS immune system struggles to manage microbiome dysbiosis and associated pathogens.

Studies of the metabolome are often conducted in an attempt to identify disease-specific biomarkers. However, the metabolome can also be screened for metabolites that directly induce or suppress biological function in patients with a given illness. These studies help dissociate cause from effect and allow for possible modulation of disease phenotype. For example, Johnson et al. (96) investigated the metabolic influence of microbial biofilms on colon cancer tissue and related cancer occurrence. They found that up-regulation of a biofilm-derived polyamide metabolite enhanced both biofilm formation and cancer growth.

## Microbes Act Differently Depending on Neighboring Species and Immune Status

Studies of microbiome activity must account for the fact that pathogens detected in patients with ME/CFS are also regularly identified in healthy subjects or in patients with related inflammatory conditions. This is particularly true of studies that have searched for EBV, HHV6, Cytomegalovirus, and other viruses able to be identified by PCR and/or antibody testing in ME/CFS cohorts. This same trend is likely to hold for less-studied or unidentified human microbes and viruses. While these “overlapping” results are often viewed as problematic, they make sense in light of research that clarifies how differently microbes act depending on host immune status, neighboring species, and a wide range of other variables. For example, susceptibility to HIV infection has been shown to vary based on the species composition and activity of the bacterial vaginal microbiome (97).

Indeed, most human microbes are pathobionts: they can change their gene expression to act as pathogens under conditions of imbalance and immunosuppression (98–100). For example, *S. pneumoniae* can persist as a highly adapted commensal or a virulent pathogen depending on its ability to evade the host immune response (101). *S. aureus* causes a range of illnesses, from skin infections to life-threatening diseases such as endocarditis and meningitis. However, ~30% of the healthy human population harbors *S. aureus* as a member of the normal nasal microbiome (102). *S. aureus* virulence in these communities is determined by a number of factors, including the signaling and competitive strategies employed by neighboring microbes.

The same is true of *Escherichia coli (E. coli)*, which also persists in numerous forms. One study found that “commensal” *E. coli* could evolve into virulent clones in fewer than 500 generations (103). For most microbes, this evolution toward pathogenicity occurs via the acquisition of new genes or alteration of the current genome in a manner that induces gene loss (104). For example, loss of *mucA* increases the ability of *Pseudomonas aeruginosa* to evade phagocytosis and resist pulmonary clearance (105).

It should also be noted that every microbial species represents many different strains, each of which may vary in the set of genes it encodes or in the copy number of such genes. This intra-species variation endows each strain with distinct functional capacities, including differences in virulence, motility, nutrient utilization, and drug resistance (106). Greenblum et al. (106) identified extensive strain-level copy-number variation across species in metagenomic samples obtained from patients with irritable bowel syndrome. This was especially true of genes tied to specific community functions, including functions related to community lifestyle. Differences in gene copy-number also impacted adaptive functions linked to obesity. Yao et al. (107) found that deletion of a single Bacteriodes gene—and the bile salt hydrolase it expresses—altered host metabolism in a manner that impacted weight management, circadian rhythm and immunity.

A microbe or a virus' location can also influence its activity. For example, much of the human population harbors HHV-6. However, in Alzheimer's disease, HHV-6A was recently identified in human brain tissue (88). There, its activity was shown capable of regulating host molecular, clinical, and neuropathological networks in a manner that can contribute to inflammation and neuronal loss. VanElzakker (108) has proposed that HHV-6 may also infect the vagus nerve in ME/CFS, resulting in altered gut-brain axis signaling in patients with the illness.

The human immune system also plays a central role in determining microbe and viral activity. A robust immune response is often capable of controlling pathogen virulence. However, if pathogens overcome the immune response, or the immune system is suppressed by medications, chemicals, or other environmental factors, pathobionts are more likely to alter their gene expression in a manner that promotes disease.

## Pathobionts Alter Their Collective Gene Expression to Drive Dysbiosis

Pathobionts can subvert the human immune response by collectively altering their gene expression. Yost et al. (100) performed an excellent gene ontology (GO) enrichment analysis of the oral microbiome during periodontal progression. Over the two-month study period, changes in the metagenome of non-progressing sites were minor. However, active sites that progressed to periodontitis were characterized by numerous functional genomic signatures. In fact, the team reported a complete rearrangement at the metagenome level between baseline sites that progressed to periodontitis and those that did not.

GO terms associated with processes including peptidoglycan biosynthesis and potassium transport were highly enriched at baseline sites that later progressed to periodontitis. Genes controlling ciliary motility and CRISPR-associated proteins were also active during initial stages of disease progression. At the breakdown point, active sites expressed genes associated with ferrous iron transport and response to oxidative stress. Progression to periodontitis was also correlated with increased expression of putative virulence factors associated with a range of bacterial species. Mycoplasma, bacteriophage, and eukaryotic viral activity were higher in progressing sites compared to baseline samples.

The team concluded that periodontitis progression is driven by the whole oral microbial community and not just a few select pathogens. In effect, under conditions of increasing inflammation and imbalance, the entire oral community appeared to *act together as a pathogen*. Indeed, groups of bacteria not generally considered pathogens upregulated a large number of the putative virulence factors in active sites. These included *Veillonella parvula*, a microbe almost always associated with dental health.

## Intracellular Pathogens Drive Microbiome Dysbiosis

Community-wide shifts in microbiome virulence are often driven by dominant pathogens—organisms that become established as central components of the microbiome while suppressing commensal growth and activity (109). In other cases, keystone pathogens promote inflammation even when present as quantitatively minor members of the microbiome. For example, *P. gingivalis* often comprises just 0.01% of periodontal biofilms, yet drives destructive changes in host-microbe interplay by profoundly impairing the innate immune response (110).

**Figure 3:**
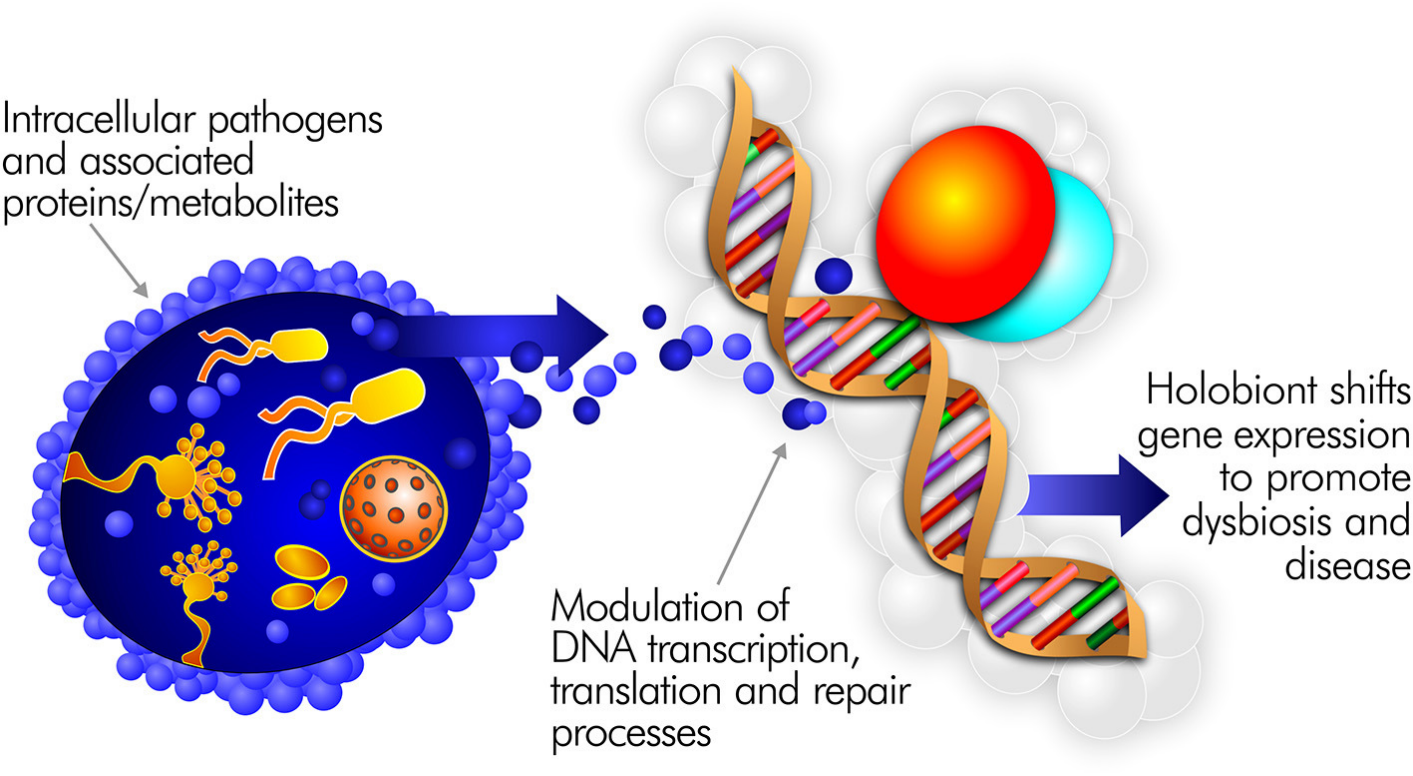
Intracellular pathogens and the proteins/metabolites they express can directly interfere with human transcription, translation, and DNA repair processes.

Pathogens able to persist inside the cells of the immune system are uniquely positioned to drive inflammatory disease (111). Indeed, most well-characterized pathogens, including many connected to ME/CFS, are capable of intracellular persistence (112). By surviving in this fashion, they can directly interfere with human transcription, translation, and DNA repair processes (Figure 3). Pathogens in the cell cytoplasm may further dysregulate the epigenetic environment (113). For example, upon infecting a macrophage, *Mycobacterium tuberculosis* alters the expression of 463 human genes (114). *H. pylori* infection predisposes to genomic instability and DNA damage, including double strand breaks (115). EBV infection of B cells can also promote persistent damage to human DNA (116).

The thousands of metabolites and proteins expressed by intracellular pathogens also interact with the host genome, further modifying human gene expression in a manner that promotes disease. Even bacterial quorum sensing peptides can dysregulate human pathway activity. Wynendaele et al. (117) found that quorum sensing molecules created by gram-negative bacteria altered human gene expression in a manner that promoted *in vitro* angiogeneisis, tumor growth, and neovascularization in colon cancer.

Intracellular pathogens can also travel between cells via recently characterized tunneling nanotubules (TNTs) (118, 119). These cytoplasmic extensions of dendritic cells, glial cells and related human cells allow for the intracellular transfer of microRNAs, messenger RNAs, prions, viruses, and even whole organelles such as mitochondria (120, 121). For example, HIV-induced tunneling nanotubule formation appears to mediate approximately half of HIV virus spread among monocyte-derived macrophages (119).

## Molecular Mimicry

Dysfunction driven by intracellular infection is compounded by the fact that microbial proteins and metabolites are often identical or similar in structure to those created by their human hosts. The molecular mimicry or sequence homology between these proteins and metabolites makes it increasingly difficult for the human holobiont to recognize “foreign” from “self.”

For example, Altindis et al. (122) found that viruses carry sequences with significant homology to human insulin-like growth factors (VILPs). These VILPs can bind human and murine IGF-1 receptors *in vitro*, resulting in autophosphorylation and downstream signaling. *E. coli* harbors a large, diverse network of proteins that actively promote endogenous DNA damage in cells (123). However, at least 280 of these DNA-damaging proteins have human homologs that also promote DNA damage and mutagenesis in the human host.

Indeed, redundancy between human and microbial metabolites, proteins, and pathways is so great that the potential for molecular mimicry to contribute to host immune and metabolic dysfunction is semi-infinite. For example, viral vesicles and human extracellular vesicles (EVs) share considerable structural and functional similarity (124). These similarities are so extensive that it is difficult to distinguish EVs from (noninfectious) viruses.

## Different Pathogens Employ Common Survival Strategies

Many pathogens employ common survival mechanisms to persist in host cells, tissue and blood. The metabolic dysfunction driven by these different microbes and viruses can result in similar clusters of human inflammatory symptoms. The ability of various pathogens to dysregulate activity of the Vitamin D Nuclear Receptor (VDR) is an excellent example of how different microbes can drive similar disease processes. The VDR regulates expression of hundreds of human genes, many of which regulate inflammatory and malignant processes (125, 126). The receptor also controls signaling of TLR2 and several families of antimicrobial peptides including cathelicidin (LL-37) (127). Pathogens capable of slowing VDR activity can subsequently facilitate their survival by slowing the innate immune response.

**Figure 4:**
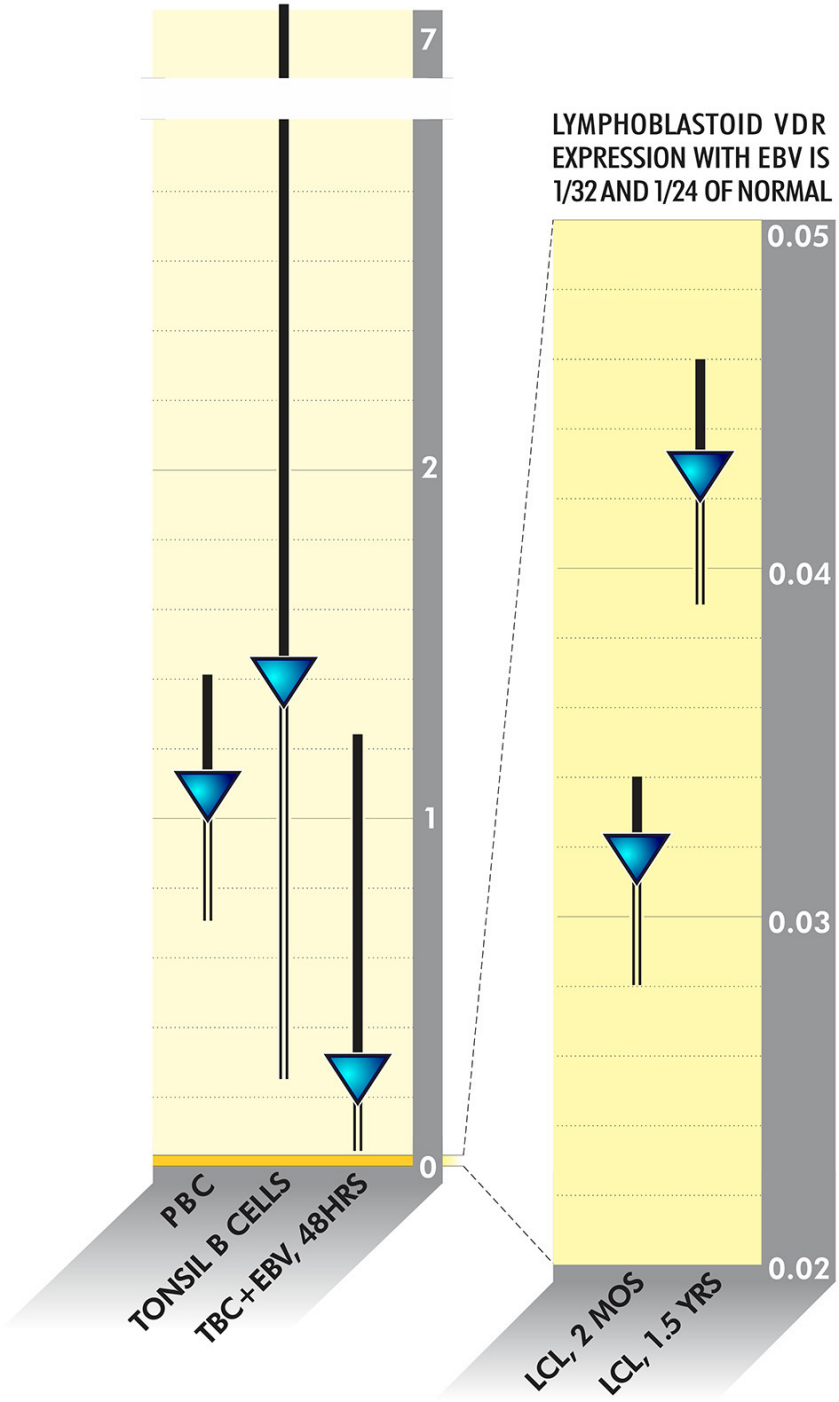
VDR activity is downregulated as much as 20 times in Epstein Barr Virus-infected lymphoblastoid cell lines (128).

Pathogens frequently linked to ME/CFS or inflammatory disease have evolved to survive in this fashion. VDR activity is downregulated as much as 20 times in Epstein Barr Virus-infected lymphoblastoid cell lines (128) (Figure 4). HIV, *M. tuberculosis* Cytomegalovirus, *Borrelia burgdorferi* and *Mycobacterium leprae* additionally dysregulate VDR activity to various degrees (114, 129–132). The fungus Aspergillus fumigatus secretes a gliotoxin that significantly downregulates VDR expression (133). Because disabling the innate immune system via the VDR pathway is such a logical survival mechanism, other uncharacterized bacteria, viruses or fungi may have also evolved to dysregulate receptor activity.

It follows that ME/CFS patients with similar symptoms may not always test positive for the same mix of pathogens and/or communities of pathobionts. Similarly, composition of the ME/CFS microbiome, proteome and metabolome should be expected to differ somewhat from study to study. Instead of worrying about these inconsistencies, the ME/CFS research community should strive to better characterize even more common mechanisms of pathogen survival and persistence.

## The Immune Response Changes in Response to Pathogen Activity

The ability of pathogens to disable the host immune response extends far beyond the VDR pathway. Pushalkar et al. (134) identified a distinct and abundant pancreatic microbiome associated with progressive pancreatic cancer. This dysbiotic microbiome drove oncogenesis by suppressing macrophage differentiation and T cell activity. Another study found that *Candida albicans*'s transition from extracellular to intracellular pathogen was accompanied by a coordinated, time-dependent shift in gene expression for both host and fungus (135). These gene expression changes led to a gradual decline in pro-inflammatory cytokine activation by the host immune system.

These findings shed light on a recent ME/CFS study. Hornig et al. (5) reported distinct alterations in plasma immune signatures—including prominent activation of both pro-and anti-inflammatory cytokines—early in the course of ME/CFS. However, these alterations were not observed in subjects with a longer duration of illness. There are a number of explanations for Hornig's observations. ME/CFS-associated pathogens may gradually alter their gene activity to persist in more latent, intracellular forms less recognized by the host immune system. Or, in early-stage ME/CFS, the immune system may actively attempt to target a growing infectious burden. Over time however, pathogens in the microbiome may disable the immune response to a point where “immune exhaustion” occurs (136, 137). Immunopathology and cytokine production would subsequently drop. The resulting disease state could be compared to a garden, in which healthy plants become progressively stifled by others, such as kudzu vine.

## “Acute” Pathogens Can Survive in Persistent Forms that Drive Chronic Symptoms

Many research teams are studying how “acute” pathogens (i.e., well-characterized agents associated with known infectious diseases) can cause chronic symptoms by persisting in latent forms. These include Zika, *Borrelia burgdorferi*, influenza, and other well-characterized viruses and bacteria (138). Zika was shown capable of persisting in the cerebrospinal fluid (CSF) and lymph nodes of infected rhesus monkeys for months after the virus had been cleared from mucosal secretions, peripheral blood and urine (139). Viral persistence in both the lymph nodes and CSF was correlated with upregulation of genes involved in pro-inflammatory and anti-apoptotic pathways.

Tens of thousands of Ebola survivors have developed chronic symptoms months or years after initial infection (140). These “Post-Ebola Syndrome” symptoms include extreme fatigue, severe pain, eye problems, and a host of neurological issues. While the virus is hard to identify in the blood of such patients, Ebola has been detected in men's semen years after “recovery” (141). Another study found that, up to a month after initial infection, influenza viruses regulated the long-term expression of inflammatory and neuron/glia-specific genes in mice (142). This was associated with chronic neuroinflammation characterized by an increase in the number of activated microglia and impairment of spatial memory formation.

Persistent measles virus is associated with conditions such as Paget's disease, multiple sclerosis and Crohn's disease (143, 144). Measles virus RNA has been detected in blood, respiratory secretions, urine, and lymphoid tissue for weeks to months after clearance of the “acute” infection (145). Doi et al. (146) found that, *in vitro*, measles virus persistence correlated with viral transition from a lytic to non-lytic mode that allowed the virus to evade the host innate immune response.

Hundreds of studies demonstrate that acute bacterial pathogens can transition into latent forms that lack a classical cell wall (1). These persistent variants are referred to as L-form bacteria. Antibiotic use can induce persistent L-form growth. In fact, researchers create L-forms by deliberately culturing classical bacteria in conjunction with the beta-lactam antibiotics (147). One microarray analysis of L-form growth revealed up-regulation of genes shared in common with persister cells and biofilms (148). L-form bacteria have been implicated in dozens of chronic conditions including rheumatoid arthritis, multiple sclerosis and sarcoidosis (1, 149, 150).

A better understanding of these chronic sequelae would greatly benefit the ME/CFS community, since a similar chronic progression of symptoms is frequently documented in ME/CFS. At different points in history, ME/CFS has been called “Post-Polio Syndrome,” “Chronic Mononucleosis Syndrome” and “Post-Viral Syndrome” due to the fact that chronic symptoms are often noted after acute infection with Polio Virus, Epstein Barr Virus, influenza or a range of other pathogens (151–153). Chia et al. (154) found that patients with acute enterovirus infection went on to develop a multitude of chronic symptoms consistent with an ME/CFS diagnosis. Years after the initial infection, enterovirus protein and RNA were still present in these patients' stomach biopsies.

It is clear that novel methodologies may be required to best identify pathogens in their latent forms. Pathogens that persist inside human immune cells and associated tunneling nanotubuoles have been particularly hard to detect. When persistent Zika virus was identified in rhesus monkeys, Zika-specific antibodies were not detected in the CSF, despite prolonged and robust responses in peripheral blood. This suggested an additional mechanism for viral persistence in certain “anotomic sanctuaries.” Modern living has also increased the likelihood that “acute” infections may generate chronic sequelae. Before the advent of antibiotics, steroid medications and childhood vaccines, almost half of all children under the age of five died from acute infectious disease (45). Today, most individuals in first-world countries survive repeated acute infections over the course of decades.

## Unique Infectious History Shapes ME/CFS Disease Progression

While certain dominant or keystone pathogens may be reliably identified in patients with ME/CFS, composition of the ME/CFS microbiome will likely differ between patients. Even in HIV/AIDS, where an easily detected virus dysregulates immunity, disease symptoms reflect a mix of those driven by HIV, and those driven by “co-infectious” agents able to take advantage of the immunocompromised host (155). No two patients with HIV/AIDS are expected to harbor the same mix of these additional persistent bacteria, fungi, and viruses. The same is true of most cancers, an increasing number of which are now tied to severe microbiome dysbiosis (34). It is widely accepted that no two cancers are alike, and any tumor-associated microbiome is expected to differ somewhat among individual study subjects (156).

This same pattern, in which unique infectious history impacts symptom presentation may also occur in ME/CFS. A recent study by Brodin et al. (45) demonstrated the profound impact of infectious history on host immunity. The team performed a systems-level analysis of 210 healthy twins between the ages of 8 and 82. They measured 204 immune parameters, including cell population frequencies, cytokine responses, and serum proteins, and found that 77% of these are dominated, and 58% almost completely determined, by non-heritable environmental influences. Many of these parameters became more variable with age, emphasizing the cumulative influence of environmental exposure.

The team also calculated how acquisition of just one chronic pathogen—cytomegalovirus (CMV)—conditions the immune response. Identical twins discordant for CMV infection showed greatly reduced correlations for many immune cell frequencies, cell signaling responses, and cytokine concentrations. In general, the influence of CMV was so broad that it affected 119 of the 204 measurements dispersed throughout the immune network. These and related findings led Brodin and team to conclude that the immune response is “very much shaped by the environment and most likely by the many different microbes an individual encounters in their lifetime.”

## Could “Successive Infection Contribute to ME/CFS?”

The above suggests that ME/CFS may be driven by a process we have termed “successive infection.” During successive infection, an “acute” infection or “initial immunosuppressive event” dysregulates the host immune system. This makes it easier for microbes to subvert the immune response by acting as polymicrobial entities. Pathobionts alter their gene expression to better promote community-wide virulence. Infected human cells fail to correctly express human metabolites in the presence of pathogen-generated proteins, metabolites, and enzymes. Dysfunction driven by molecular mimicry increases. Certain pathogens may infect mitochondria or central nervous system tissue. Intracellular pathogens slow the human immune response, causing the host to more easily acquire other infectious agents. This creates a snowball effect in which the microbiome becomes increasingly dysbiotic as the strength of the immune response weakens over time.

Eventually, the human host may present with symptoms characteristic of ME/CFS or a related inflammatory diagnosis. The unique symptoms any one patient develops are expected to vary based on the species, strain, virulence, and location of the pathogens driving dysbiosis, along with the myriad ways in which the metabolites and proteins created by these organisms cause dysfunction by interacting with those of the host.

Early childhood infections may predispose to dysbiosis at a later date (157). For example, measles depletes host B and T lymphocytes for up to 2–3 years after initial infection. This immunosuppression can reset previously acquired immunity and renders the host more susceptible to other pathogens (158). In other cases, a toxic environmental exposure, infection during surgery or the difficulty of enduring a traumatic event may weaken the immune response to a point where previously subclinical infections become active. ME/CFS outbreaks, in which numerous patients developed the illness at relatively the same time, may well represent this phenomenon at work.

The successive infectious process may even begin in the womb. Depending on the health of the parent, founding microbiome communities in the placenta, vagina and breast milk may already be dysbiotic. Cabrera-Rubio et al. (159) found that the breast milk microbiome of obese mothers tended to contain a different and less diverse bacterial community then that of normal-weight mothers. Pathogens in the placental microbiome can alter methylation of human DNA in a manner that may negatively impact the later life disease of premature babies (160).

Many aspects of modern living can additionally drive successive infection. Antibiotic use disrupts the ecology of the human microbiome (161). Antibiotic resistance genes from farm animals and produce are regularly transferred into the human food supply (162). Electromagnetic radiation has been shown to lower immunity (163). The immunosuppressive biologics and supplements often prescribed for inflammatory disease further allow pathobionts in the microbiome to proliferate. For example, Diaz et al. (164) found that the salivary bacteriome of patients taking immunosuppressive biologics was more permissive to the growth of oral opportunistic pathogens.

Modern society also exposes the average person to pathogens our recent ancestors were unlikely to encounter. International airports harbor pathogens from across the globe (165). Food products, and the microbes they contain, are frequently imported from foreign destinations (166). One study found a range of opportunistic pathogens enriched in showerhead biofilms (167). Numerous pathogens were identified in commonly smoked cigarettes (168). Many such pathogens are capable of persisting in human microbiome ecosystems, where the host may lack immunity toward their presence.

## ME/CFS Patients Should be Studied in Concert With Related Inflammatory Conditions

ME/CFS is a spectrum disorder with a diagnostic criterion that includes a range of physical and neurological symptoms (2). If successive infection contributes to ME/CFS, this variability in symptom presentation is expected. Furthermore, factoring “unique infectious history” into the disease process helps explain why patients with ME/CFS often suffer from a multitude of symptoms not included in the official diagnostic criteria.

Because patients with ME/CFS suffer from such diverse symptoms, it has been argued that they should be grouped into separately studied “subgroups” (169). In some cases this makes sense. For example, studies that distinguish early-stage/late-stage ME/CFS patients may further elucidate how the immune response is modified by the microbiome over time. However, if successive infection contributes to ME/CFS, future research should also focus on better understanding the common pathogenesis shared by all subjects.

Indeed, successive infection may contribute to other conditions tied to microbiome dysbiosis, persistent infection, and adverse environmental exposure. ME/CFS should be studied in concert with these other conditions, which include Gulf War Syndrome, Ehlers-Danlos Syndrome, Chronic Lyme Disease, and fibromyalgia among others (170–174). The high levels of comorbidity and symptom overlap between patients with ME/CFS and these related inflammatory diagnoses strengthens this assumption.

## “Autoantibodies” in ME/CFS Are Likely Created in Response to Persistent Pathogens

A number of autoantibodies have been detected in patients with ME/CFS (4). This has led some research teams to postulate that ME/CFS should be regarded as an “autoimmune” disorder (175). However, the classical “theory of autoimmunity” is in the process of being re-evaluated (78, 176). Increasing evidence suggests that “autoantibodies” are actually created in response to chronic, persistent microbiome pathogens. Under such conditions molecular mimicry or structural homology between pathogen and host proteins can result in “collateral damage” toward human tissue. For example, Vojdani et al. (177) found that 25 different Alzheimer's-associated pathogens or their molecules could react with antibodies against amyloid beta via molecular mimicry or the binding of bacterial toxins to amyloid beta.

A growing body of research documents “autoantibody” production in response to a range of bacterial, viral and fungal pathogens/pathobionts. These pathogens are not short-term “triggers” but persist as members of complex microbiome communities. For example, “autoantibody” production was recently tied to microbiome pathobiont *Enterococcus gallinarum*. Manfredo et al. (178) detected *E. galinarum* in the mesenteric veins, lymph nodes, spleens and livers of mice made genetically prone to autoimmunity. In these mice, the bacterium initiated the production of “autoantibodies,” inflammation and activated T cells. However, this “autoantibody” production stopped when *E. gallinarum's* growth was suppressed with the antibiotic vancomycin or with an intramuscular vaccine. In addition, *E. gallinarum*–specific DNA was recovered from liver biopsies of human autoimmune patients, and co-cultures with human hepatocytes replicated the murine findings.

Indeed, many pathogens can drive the activated or clonal T cell expansion often associated with “autoimmune” conditions. For example, Tuffs et al. (179) found that *S. aureus* endotoxins triggered uncontrolled activation of T cells. This led to a pro-inflammatory cytokine storm that accounted for both T cell activation and related inflammation.

## The Gut-Brain Axis and the Brain Microbiome

Microbes and their metabolites control bidirectional signaling between the gut and the brain via pathways collectively known as the gut-brain axis (180). The gut-brain axis involves various afferent and efferent pathways including the vagus nerve, with signaling impacting neural, endocrine, and immune processes. The gut's enteric nervous system contains over 100 million neurons—more than in either the peripheral nervous system or spinal cord (181). It follows that gut microbiome dysbiosis may modulate brain activity. For example, Sampson et al. (79) transplanted fecal samples from patients with Parkinson's disease into germ-free mice. These mice, and not controls, exhibited physical symptoms associated with Parkinson's.

However, there is also growing evidence that humans may harbor a brain microbiome. Researchers at Harvard University are characterizing this ecosystem as part of the ongoing “Brain Microbiome Project.” In what marks a major paradigm shift, multiple research teams have shown that both amyloid beta and prion protein (PrP) are potent antimicrobial peptides (182, 183). Amyloid beta exhibited antimicrobial activity against a range of common microorganisms with a potency equivalent to, and in some cases greater than, cathelicidin (LL-37) (184). These pathogens included *S. typhimurium, Candida albicans* and, more recently, a number of herpesviruses capable of persisting in the Alzheimer's brain (88, 185).

The findings strongly suggest that amyloid beta and PrP are not useless byproducts of abnormal brain catabolism. Rather, they appear to form a mediated response of the innate immune system toward infectious agents in brain tissue (186). A number of brain abnormalities have been reported in patents with ME/CFS (187). These findings must be interpreted in light of these novel infection-based paradigms and in concert with emerging data on the brain microbiome.

## What About the Human Genome?

In a study of aortic aneurysms, Gottlieb et al. (188) reported that BAK1 SNP-containing alleles were detected in aortic tissue but not in blood samples from the same patients. More recently Ursini et al. (189) found that schizophrenia gene risk loci that interact with early-life complications are highly expressed in the placenta. However, these loci were differentially expressed in placentas from women who suffered complications during pregnancy. They were also differentially upregulated in placentae from male compared with female offspring.

These and related findings strongly suggest that the environment can select for human genome activity. For example, Harley et al. (190) found that in EBV infected cells, EBNA2 and its transcription factors modulated the activity of human genes associated with risk for multiple sclerosis, rheumatoid arthritis, type 1 diabetes and other conditions. In fact, nearly half of systemic lupus erythematosus risk loci were occupied by EBNA2 and co-clustering human transcription factors.

Studies of the human genome must also account for the full extent of microbial DNA and RNA in human tissue and blood. If a genomic assembler fails to account for this contamination, chances of a false positive single nucleotide polymorphism (SNP) increase significantly during analysis (191). Contamination with even a small amount of microbial DNA/RNA—just one or two base pairs of difference—is enough to cause significant statistical errors in this fashion.

## Immunostimulation in the Treatment of ME/CFS

If ME/CFS is driven by successive infection, treatments that support or activate the human immune system could improve microbiome health by allowing patients to better target persistent pathogens. Development of such therapies should be a priority for the ME/CFS research community. However, most immunostimulative treatments that target pathogens are characterized by immunopathology—a cascade of reactions in which inflammation, cytokine release and endotoxin release are generated as part of the immune system's response to microbial death (192–194). The death of intracellular pathogens is particularly difficult for the host to manage, as the body must deal with debris generated from apoptosis. Inflammation is also generated in response to bacterial cell wall components including the endotoxin lipopolysaccharide of Gram-negative strains (192). Luckily, immunopathology-generated symptoms are generally temporary in nature, and tend to subside as an increasing number of pathogens are eradicated.

Temporary immunopathology resulting from antimicrobial treatment has been documented for over a century, with symptoms varying depending on the nature of targeted pathogens. First referred to as the Jarisch–Herxheimer reaction, the phenomenon was originally observed during treatment of syphilis with mercury and penicillin (195, 196). The Jarisch-Herxheimer reaction/immunopathology has since been noted in a broad spectrum of chronic inflammatory conditions including tuberculosis and Brucellosis (197). Short-term immunopathology is also a central feature of acute infection. If a patient develops the flu, inflammatory symptoms increase as the immune system releases cytokines and chemokines in response to the infecting virus.

HIV/AIDS patients undergo a form of immunopathology called Immune Reconstitution Inflammatory Syndrome (IRIS) following treatment with Combination Antiretroviral Therapy (ART) (198). IRIS occurs as ART enables the host immune system to better target pathogens acquired during previous periods of HIV-driven immunosuppression. A range of well-characterized pathogens have been linked to IRIS including the herpesviruses and *M. tuberculosis* (193). However, the inflammatory reaction is also noted in culture-negative patients, suggesting IRIS may also involve novel or uncharacterized pathogens (198).

Over the past decade, in concert with our clinical collaborators, we developed an immunostimulative therapy used to treat patients with a range of chronic inflammatory conditions (200). Treatment centers on the use of a putative VDR agonist in the form of olmesartan medoxomil, with the goal of reactivating components of innate immunity under VDR control. In 2013, we published a series of case histories demonstrating improvement in ME/CFS patients administered this treatment (199). However, all ME/CFS subjects administered the therapy experienced immunopathology and associated inflammatory symptom increases that lasted for many years. As a general trend, patients administered the treatment during earlier stages of disease experienced less immunopathology, emphasizing the need for immunostimulative therapies to be used in a predictive and even preventative fashion.

While some ME/CFS physicians may feel uneasy about the suffering induced by immunopathology, other research communities have become accustomed to treatments that cause temporary discomfort. Novel cancer immunotherapies also generate immunopathology by activating patient T cells. This allows the immune system to better target malignant tumors (194, 201). The resulting “cytokine release syndrome” leads to profound, temporary, symptom increases. However, these increased symptoms are considered acceptable, as patients who endure the reaction are more likely to enter a state of remission. Since many forms of cancer are tied to severe microbiome dysbiosis, at least part of the “cytokine release syndrome” may result from the death of persistent pathogens.

## Discussion

The history of ME/CFS strongly suggests that infectious agents play a central role in driving the disease process. However, the discovery of the human microbiome has revolutionized the manner in which persistent infection and chronic inflammation are understood and studied. Humans harbor extensive microbiome communities of bacteria, viruses, and fungi in nearly all tissue and blood. The hundreds of millions of unique genes harbored by this microbiome dwarf the ~20,500 in the human genome. Humans are best described as holobionts, in which these microbial genomes and the human genome continually interact to regulate host gene expression, metabolism and immunity.

Many inflammatory disease states, including neurological conditions and cancers, are tied to dysbiosis or imbalance of human microbiome communities in various body sites. While gut microbiome dysbiosis has already been identified in ME/CFS, distinct microbial and viral communities may additionally persist in ME/CFS blood and brain tissue. Possible identification of these microbiomes should be a priority for the ME/CFS research community, but analysis requires the use of very specific technologies and methodologies.

Pathogens and their associated proteins/metabolites control human metabolism and gene expression in a manner that can push the human holobiont toward a state of illness. Studies of microbiome dysbiosis in ME/CFS must consider this microbe and viral activity. Most human microbes can alter their gene expression to act as pathogens under conditions of imbalance and immunosuppression. This pathobiont behavior is further determined by the activity and virulence of neighboring microbes. Patients with ME/CFS may harbor many of the same microbes and viruses as heathy individuals, yet these pathobionts may act with increased virulence in patients with the illness.

Intracellular pathogens, including several associated with ME/CFS, have been shown to directly interfere with human transcription, translation, and DNA repair processes. Molecular mimicry between host and pathogen proteins/metabolites further exacerbates this interference. Interacting microbes can also drive disease by changing their collective gene expression. Other pathogens disable mitochondria, or may infect central nervous system tissue in ways that dysregulate signaling via the gut-brain axis. Antibodies and/or clonal T cells identified in patients with ME/CFS are likely activated in response to many of these persistent microbiome pathogens.

Different pathogens have evolved similar survival mechanisms to disable the host immune response and/or host metabolic pathways. The metabolic dysfunction driven by these different microbes can result in similar clusters of inflammatory symptoms. ME/CFS may be driven by this pathogen-induced dysfunction, with the nature of dysbiosis and symptom presentation varying based on a patient's unique infectious and environmental history. An initial infection or environmental exposure weakens the host immune system. This makes it easier for pathobionts to subvert the immune response and interfere with host gene expression and metabolism. A snowball effect begins, in which the microbiome becomes increasingly dysbiotic as the strength of the immune response weakens over time. The unique symptoms any one ME/CFS patient develops are expected to vary depending on the location, species, strain and virulence of the pathogens driving this dysbiosis. Thus, while certain dominant or keystone pathogens may be identified in ME/CFS, composition of the ME/CFS microbiome, metabolome, and proteome should be expected to differ somewhat among individual patients.

These common mechanisms suggest that ME/CFS is best studied in concert with other chronic conditions tied to microbiome dysbiosis, persistent infection and adverse environmental exposure. These include fibromyalgia and Gulf War Syndrome, but also conditions like Post-Ebola Syndrome in which severe chronic symptoms develop after infection with an “acute” infectious agent that is able to persist in latent forms.

Treatments that support or activate the human immune system could allow ME/CFS patients to improve microbiome health by better targeting pathogens over time. Like the novel immunotherapies being developed for cancers, these immunostimulative therapies would be expected to generate temporary immunopathology. Institutional reviewers hesitant to approve immunopathology-based therapies should consider that ME/CFS quality of life is typically very low, with patients demonstrating a substantial increase in mortality from suicide (202).

It often takes patients years to receive a diagnosis of ME/CFS. This delay wastes a valuable period during which the immune system is most responsive to immunostimulatory treatment. Patients treated during earlier stage disease are also less likely to experience severe or long-lasting immunopathology. This suggests that immunostimulative therapies should be administered in a predictive and even preventative fashion. In addition, interventions or treatments that might help patients better manage the byproducts of immunopathology (bacterial LPS etc.) should become a priority for the research community.

The overall success of ME/CFS research also hinges on the scientific community's willingness to embrace the concept of the human holobiont. In ME/CFS, the immune response, metabolism, central nervous system, and human gene expression are all linked by the activity of the microbiome and its associated proteins/metabolites. A greater focus on these interconnected systems is necessary, which will require increased collaboration between separate research teams.

## Author Contributions

AP drafted the manuscript, analyzed and interpreted data, and revised the manuscript for critically important intellectual content. TM drafted parts of the manuscript, analyzed/interpreted data and revised the manuscript for critically important intellectual content.

### Conflict of Interest Statement

The authors declare that the research was conducted in the absence of any commercial or financial relationships that could be construed as a potential conflict of interest.
